# Stochastic Mirror Descent Dynamics and Their Convergence in Monotone Variational Inequalities

**DOI:** 10.1007/s10957-018-1346-x

**Published:** 2018-07-18

**Authors:** Panayotis Mertikopoulos, Mathias Staudigl

**Affiliations:** 1 CNRS, Inria, LIG, Univ. Grenoble Alpes, 38000 Grenoble, France; 2 Department of Quantitative Economics, School of Business and Economics, Maastricht University, P.O. Box 616, 6200 MD Maastricht, The Netherlands

**Keywords:** Mirror descent, Variational inequalities, Saddle-point problems, Stochastic differential equations, 90C25, 90C33, 90C47

## Abstract

We examine a class of stochastic mirror descent dynamics in the context of monotone variational inequalities (including Nash equilibrium and saddle-point problems). The dynamics under study are formulated as a stochastic differential equation, driven by a (single-valued) monotone operator and perturbed by a Brownian motion. The system’s controllable parameters are two variable weight sequences, that, respectively, pre- and post-multiply the driver of the process. By carefully tuning these parameters, we obtain global convergence in the ergodic sense, and we estimate the average rate of convergence of the process. We also establish a large deviations principle, showing that individual trajectories exhibit exponential concentration around this average.

## Introduction

Dynamical systems governed by monotone operators play an important role in the fields of optimization (convex programming), game theory (Nash equilibrium and generalized Nash equilibrium problems), fixed point theory, partial differential equations and many other fields of applied mathematics. In particular, the study of the relationship between continuous- and discrete-time models has given rise to a vigorous literature at the interface of these fields—see, e.g., [1] for a recent overview and [2] for connections to accelerated methods.

The starting point of much of this literature is that an iterative algorithm can be seen as a discretization of a continuous dynamical system. Doing so sheds new light on the properties of the algorithm, it provides Lyapunov functions, which are useful for its asymptotic analysis, and often leads to new classes of algorithms altogether. A classical example of this arises in the study of (projected) gradient descent dynamics and its connection with Cauchy’s steepest descent algorithm—or, more generally, in the relation between the mirror descent (MD) class of algorithms [3] and dynamical systems derived from Bregman projections and Hessian Riemannian metrics [4–6].

## Problem Formulation and Related Literature

Throughout this paper, $\mathcal {X}$ denotes a compact and convex subset of an *n*-dimensional real space $\mathcal {V}\cong \mathbb {R}^n$ with norm $||\cdot ||$. We will also write $\mathcal {Y}\equiv \mathcal {V}^{*}$ for the dual of $\mathcal {V}$, $\langle y,x\rangle$ for the canonical pairing between $y\in \mathcal {V}^{*}$ and $x\in \mathcal {V}$, and $||y||_{*}:=\sup \{\langle y,x\rangle :||x|| \le 1\}$ for the dual norm of *y* in $\mathcal {V}^{*}$. We denote the relative interior of $\mathcal {X}$ by ${{\mathrm{ri}}}(\mathcal {X})$, and its boundary by ${{\mathrm{bd}}}(\mathcal {X})$.

In this paper, we are interested in deriving dynamical system approaches to solve monotone variational inequalities (VIs). To define them, let $v:\mathcal {X}\rightarrow \mathcal {Y}$ be a Lipschitz continuous monotone map, i.e.,

$$\begin{aligned} {\mathrm{(H1)}}\qquad ||v(x)-v(x')||_{*} \le L ||x-x'|| \quad \text {and} \quad \langle v(x)-v(x'),x-x'\rangle \ge 0, \end{aligned}$$

for some $L>0$ and all $x,x'\in \mathcal {X}$.

Throughout this paper, we will be interested in solving the Minty VI:

MVI $$\begin{aligned} \text {Find } x_{*}\in \mathcal {X}\text { such that } \langle v(x),x-x_{*}\rangle \ge 0 \text { for all } x\in \mathcal {X}. \end{aligned}$$

Since *v* is assumed continuous and monotone, this VI problem is equivalent to the *Stampacchia* VI:^1^

SVI $$\begin{aligned} \text {Find } x_{*}\in \mathcal {X}\text { such that } \langle v(x_{*}),x-x_{*}\rangle \ge 0 \text { for all } x\in \mathcal {X}. \end{aligned}$$

When we need to keep track of $\mathcal {X}$ and *v* explicitly, we will refer to (MVI) and/or (SVI) as ${{\mathrm{VI}}}(\mathcal {X},v)$. The solution set of ${{\mathrm{VI}}}(\mathcal {X},v)$ will be denoted as $\mathcal {X}_{*}$; by standard results, $\mathcal {X}_{*}$ is convex, compact and nonempty [9]. Below, we present a selected sample of examples and applications of VI problems; for a more extensive discussion, see [9–11].

### Example 2.1

(Convex optimization) Consider the problem

Opt $$\begin{aligned} \min _{x\in \mathcal {X}}f(x), \end{aligned}$$

where $f:\mathcal {X}\rightarrow \mathbb {R}$ is convex and continuously differentiable on $\mathcal {X}$. If $x_{*}$ is a solution of (Opt), first-order optimality gives

$$\begin{aligned} \langle \nabla f(x_{*}),x-x_{*}\rangle \ge 0 \quad \text {for all } x\in \mathcal {X}. \end{aligned}$$

Since *f* is convex, $v= \nabla f$ is monotone, so (Opt) is equivalent to ${{\mathrm{VI}}}(\mathcal {X},\nabla f)$ [12].

### Example 2.2

(Saddle-point problems) Let $\mathcal {X}^{1}\subseteq \mathbb {R}^{n_{1}}$ and $\mathcal {X}^{2}\subseteq \mathbb {R}^{n_{2}}$ be compact and convex, and let $U:\mathcal {X}^{1}\times \mathcal {X}^{2}\rightarrow \mathbb {R}$ be a smooth convex-concave function (i.e., $U(x^{1},x^{2})$ is convex in $x^{1}$ and concave in $x^{2}$). Then, the associated *saddle-point* (or *min-max*) problem is to determine the *value* of *U*, defined here as

Val $$\begin{aligned} {{\mathrm{\mathsf {val}}}}= \min _{x^{1}\in \mathcal {X}^{1}} \max _{x^{2}\in \mathcal {X}^{2}} U(x^{1},x^{2}) = \max _{x^{2}\in \mathcal {X}^{2}} \min _{x^{1}\in \mathcal {X}^{1}} U(x^{1},x^{2}). \end{aligned}$$

Existence of ${{\mathrm{\mathsf {val}}}}$ follows directly from von Neumann’s minimax theorem. Moreover, letting

1 $$\begin{aligned} v(x^{1},x^{2}):= \big (\nabla _{x^{1}} U(x^{1},x^{2}), - \nabla _{x^{2}} U(x^{1},x^{2}) \big ), \end{aligned}$$

it is easy to check that *v* is monotone as a map from $\mathcal {X}:=\mathcal {X}^{1}\times \mathcal {X}^{2}$ to $\mathbb {R}^{n_{1}+n_{2}}$ (because *U* is convex in its first argument and concave in the second). Then, as in the case of (Opt), first-order optimality implies that the saddle-points of (Val) are precisely the solutions of ${{\mathrm{VI}}}(\mathcal {X},v)$ [13].

### Example 2.3

(Convex games) One of the main motivations for this paper comes from determining Nash equilibria of games with convex cost functions. To state the problem, let $\mathcal {N} := \{1,\cdots ,N\}$ be a finite set of *players* and, for each $i\in \mathcal {N}$, let $\mathcal {X}^{i}\subseteq \mathbb {R}^{n_{i}}$ be a compact convex set of *actions* that can be taken by player *i*. Given an action profile $x = (x^{1},\cdots ,x^{N}) \in \mathcal {X}:=\prod _{i} \mathcal {X}^{i}$, the cost for each player is determined by an associated *cost function* $c^{i}:\mathcal {X}\rightarrow \mathbb {R}$. The unilateral minimization of this cost leads to the notion of Nash equilibrium, defined here as an action profile $x_{*}= (x_{*}^{i})_{i\in \mathcal {N}}$ such that

NE $$\begin{aligned} c^{i}(x_{*}) \le c^{i}(x^{i};x_{*}^{-i}) \quad \text {for all } x^{i}\in \mathcal {X}^{i}, i\in \mathcal {N}. \end{aligned}$$

Of particular interest to us is the case, where each $c^{i}$ is smooth and individually convex in $x^{i}$. In this case, the profile $v(x) = (v^{i}(x))_{i\in \mathcal {N}}$ of individual gradients $v^{i}(x):= \nabla _{x^{i}} c^{i}(x)$ forms a monotone map and, by first-order optimality, the Nash equilibrium problem (NE) boils down to solving ${{\mathrm{VI}}}(\mathcal {X},v)$ [9, 14].

In the rest of this paper, we will consider two important special cases of operators $v:\mathcal {X}\rightarrow \mathcal {V}^{*}$, namely:

1. *Strictly monotone* problems, i.e., when
  $$\begin{aligned} \langle v(x') - v(x),x'-x\rangle \ge 0 \qquad \qquad \qquad \text {with equality if and only if } x=x'. \end{aligned}$$
2. *Strongly monotone* problems, i.e., when
  $$\begin{aligned} \langle v(x')-v(x),x'-x\rangle \ge \gamma ||x'-x||^{2} \quad \text {for some } \gamma >0. \end{aligned}$$

Clearly, strong monotonicity implies strict monotonicity, which, in turn, implies ordinary monotonicity. In the case of convex optimization, strict (respectively, strong) monotonicity corresponds to strict (respectively, strong) convexity of the problem’s objective function. Under either refinement, (MVI) admits a unique solution, which will be referred to as “the” solution of (MVI).

### Contributions

Building on the above, this paper is concerned with a *stochastic* dynamical system resulting from Nesterov’s well-known “dual-averaging” mirror descent algorithm [13], perturbed by noise, and/or random disturbances. Heuristically, this algorithm aggregates descent steps in the problem’s (unconstrained) dual space, and then “mirrors” the result back to the problem’s feasible region to obtain a candidate solution at each iteration. This “mirror step” is performed as in the classical setting of [3, 4], but the dual aggregate is further post-multiplied by a variable parameter (thus turning “dual aggregates” into “dual averages”). Thanks to this averaging, the resulting algorithm is particularly suited for problems, where only noisy information is available to the optimizer, rendering it particularly useful for machine learning and engineering applications [15], even when the stochastic environment is not stationary [16].^2^

In more detail, the dynamics under study are formulated as a stochastic differential equation (SDE) driven by a (single-valued) monotone operator and perturbed by an Itô martingale noise process. As in Nesterov’s original method [13], the dynamics’ controllable parameters are two variable weight sequences, that, respectively, pre- and post-multiply the drift of the process: The first acts as a “step-size” of sorts, whereas the second can be seen as an “inverse temperature” parameter (as in simulated annealing). By carefully tuning these parameters, we are then able to establish the following results: First, if the intensity of the noise process decays with time, the dynamics converge to the (deterministic) solution of the underlying VI (cf. Sect. 4.2). Second, in the spirit of the ergodic convergence analysis of [13], we establish that this convergence can be achieved at an $\mathcal {O}(1/\sqrt{t})$ rate on average (Sect. 4.3).^3^ Finally, in Sect. 4.4, we establish a large deviations principle showing that, as far as ergodic convergence is concerned, the above convergence rate holds with (exponentially) high probability, not only in the mean.

Conceptually, our work has close ties to the literature on dynamical systems that arise in the solution of VIs, see, e.g., [2, 4, 19–22], and references therein. More specifically, a preliminary version of the dynamics considered in this paper was recently studied in the context of convex programming and gradient-like flows in [23, 24]. The ergodic part of our analysis here extends the results of [24] to saddle-point problems and monotone variational inequalities, while the use of two variable weight sequences allows us to obtain almost sure convergence results without needing to rely on a parallel-sampling mechanism for variance reduction as in [23].

### Stochastic Mirror Descent Dynamics

Mirror descent is an iterative optimization algorithm combining first-order oracle steps with a “mirror step” generated by a projection-type mapping. For the origins of the method, see [3]. The key ingredient defining this mirror step is a generalization of the Euclidean distance known as a “distance-generating” function:

#### Definition 2.1

We say that $h:\mathcal {X}\rightarrow \mathbb {R}$ is a *distance-generating function* on $\mathcal {X}$, if

(a): *h* is *continuous*.
(b): *h* is *strongly convex*, i.e., there exists some $\alpha >0$ such that
  $$\begin{aligned} h(\lambda x+(1-\lambda )x') \le \lambda h(x)+(1-\lambda )h(x')-\frac{\alpha }{2}\lambda (1-\lambda )||x-x'||^{2}, \end{aligned}$$
  for all $x,x'\in \mathcal {X}$ and all $\lambda \in [0,1]$.

Given a distance-generating function on $\mathcal {X}$, its convex conjugate is given by

$$\begin{aligned} h^{*}(y):= \max _{x\in \mathcal {X}} \{\langle y,x\rangle - h(x) \}, \quad y\in \mathcal {Y}, \end{aligned}$$

and the induced *mirror map* is defined as

$$\begin{aligned} Q(y):= {\mathop {{{\mathrm{argmax}}}}\limits _{x\in \mathcal {X}}} \{ \langle y,x\rangle - h(x) \}. \end{aligned}$$

Thanks to the strong convexity of *h*, *Q*(*y*) is well-defined and single-valued for all $y\in \mathcal {Y}$. In particular, as illustrated in the examples below, it plays a role similar to that of a projection mapping:

#### Example 2.4

(Euclidean distance) If $h(x) = \frac{1}{2} ||x||_{2}^{2}$, then the induced mirror map is the standard Euclidean projector

$$\begin{aligned} Q(y) = {\mathop {{{\mathrm{argmax}}}}\limits _{x\in \mathcal {X}}} \left\{ \sum \nolimits _{j=1}^{n} y_{j} x_{j} - \frac{1}{2} \sum \nolimits _{j=1}^{n} x_{j}^{2} \right\} = {\mathop {{{\mathrm{argmin}}}}\limits _{x\in \mathcal {X}}} ||x-y||_{2}^{2}. \end{aligned}$$

#### Example 2.5

(Gibbs–Shannon entropy) If $\mathcal {X}= \{x\in \mathbb {R}_{+}^{n}:\sum _{j=1}^{n} x_{j}=1\}$ is the unit simplex in $\mathbb {R}^n$, then the (negative) Gibbs–Shannon entropy $h(x) = \sum _{j=1}^{n} x_{j} \log x_{j}$ gives rise to the so-called *logit choice* map

$$\begin{aligned} Q(y) = \frac{(\exp (y_{j}))_{j=1}^{n}}{\sum _{k=1}^{n} \exp (y_{k})}. \end{aligned}$$

#### Example 2.6

(Fermi–Dirac entropy) If $\mathcal {X}= [0,1]^{n}$ is the unit cube in $\mathbb {R}^n$, then the (negative) Fermi–Dirac entropy $h(x) = \sum _{j=1}^{n} [x_{j} \log (x_{j}) + (1-x_{j})\log (1-x_{j})]$ induces the so-called *logistic map*

$$\begin{aligned} Q(y) = \left( \frac{\exp (y_{j})}{1 + \exp (y_{j})}\right) _{j=1}^{n}. \end{aligned}$$

For future reference, some basic properties of mirror maps are collected below:

#### Proposition 2.1

Let *h* be a distance-generating function on $\mathcal {X}$. Then, the induced mirror map $Q:\mathcal {Y}\rightarrow \mathcal {X}$ satisfies the following properties:

- $x=Q(y)$ if and only if $y\in \partial h(x)$, where
  $$\begin{aligned} \partial h(x):=\{p\in \mathcal {V}^{*} : h(y)\ge h(x)+\langle p,y-x\rangle \quad \forall y\in \mathcal {X}\}, \end{aligned}$$
  is the subgradient of *h* at *x*. In particular, $\mathrm{im}\,Q=\mathrm{dom}\,\partial h=\{x\in \mathcal {X}: \partial h(x)\ne \varnothing \}$.
- $h^{*}$ is continuously differentiable on $\mathcal {Y}$ and $\nabla h^{*}(y) = Q(y)$ for all $y\in \mathcal {Y}$.
- $Q(\cdot )$ is $(1/\alpha )$-Lipschitz continuous.

The properties reported above are fairly standard in convex analysis; for a proof, see, e.g., [12, Theorem 12.60(b)]. Of particular importance is the identity $\nabla h^{*} = Q$, which provides a quick way of calculating *Q* in “prox-friendly” geometries (such as the examples discussed above).

Now, as mentioned above, mirror descent exploits the flexibility provided by a (not necessarily Euclidean) mirror map by using it to generate first-order steps along *v*. For concreteness, we will focus on the so-called “dual averaging” variant of mirror descent [13], defined here via the recursion

2 $$\begin{aligned} y_{t+1} = y_{t} - \lambda _{t} v(x_{t}), \qquad x_{t+1} = Q(\eta _{t+1}y_{t+1}), \end{aligned}$$

where:

1. $t=0,1,\cdots$ denotes the stage of the process.
2. $y_{t}$ is an auxiliary dual variable, aggregating first-order steps along *v*.^4^
3. $\lambda _{t}$ is a variable step-size parameter, *pre-multiplying* the input at each stage.
4. $\eta _{t}$ is a variable weight parameter, *post-multiplying* the dual aggregate $y_{t}$.^5^

Passing to continuous time, we obtain the *mirror descent dynamics*

MD $$\begin{aligned} \mathrm{d}y(t) = -\lambda (t)\, v(x(t)) \,\mathrm{d}t, \qquad x(t) = Q(\eta (t) y(t)), \end{aligned}$$

with $\eta (t)$ and $\lambda (t)$ serving the same role as before (but now defined over all $t\ge 0$). In particular, our standing assumption for the parameters $\lambda$ and $\eta$ of (MD) will be that

$$\begin{aligned} {\mathrm{(H2)}}\qquad \eta (t) \text { and } \lambda (t) \text { are positive, } C^{1}\text {-smooth and nonincreasing.} \end{aligned}$$

Heuristically, the assumptions above guarantee that the dual process *y*(*t*) does not grow too large too fast, so blow-ups in finite time are not possible. Together with the basic convergence properties of the dynamics (MD), this is discussed in more detail in Sect. 3 below.

The primary case of interest in our paper is when the oracle information for *v*(*x*) in (MD) is subject to noise, measurement errors and/or other stochastic disturbances. To account for such perturbations, we will instead focus on the *stochastic mirror descent dynamics*

SMD $$\begin{aligned} \mathrm{d}Y(t) = -\lambda (t)\, \left[ v(X(t)) \,\mathrm{d}t + \,\mathrm{d}M(t)\right] , \qquad X(t) = Q(\eta (t)Y(t)) \end{aligned}$$

where *M*(*t*) is a continuous martingale with respect to some underlying stochastic basis $(\varOmega ,\mathcal {F},(\mathcal {F}_{t})_{t\ge 0},\mathbb {P})$.^6^ In more detail, we assume for concreteness that the stochastic disturbance term *M*(*t*) is an Itô process of the form

$$\begin{aligned} dM(t) = \sigma (X(t),t) \cdot \,\mathrm{d}W(t), \end{aligned}$$

where *W*(*t*) is a *d*-dimensional Wiener process adapted to $\mathcal {F}_{t}$, and $\sigma (x,t)$ is an $n\times d$ matrix capturing the *volatility* of the noise process. Heuristically, the volatility matrix of *M*(*t*) captures the intensity of the noise process, and the possible correlations between its components.

In terms of regularity, we will be assuming throughout that $\sigma (x,t)$ is measurable in *t*, as well as bounded, and Lipschitz continuous in *x*. Formally, we posit that there exists a constant $\ell >0$ such that

$$\begin{aligned} {\mathrm{(H3)}}\qquad \sup \nolimits _{x,t} ||\sigma (x,t)|| < \infty \quad \text {and} \quad ||\sigma (x',t) - \sigma (x,t)|| \le \ell ||x'-x||, \end{aligned}$$

where

$$\begin{aligned} ||\sigma || := \sqrt{{{\mathrm{tr}}}\left[ \sigma \sigma ^{\top }\right] } = \sqrt{\sum \nolimits _{i=1}^{n} \sum \nolimits _{j=1}^{d} |\sigma _{ij}|^{2}} \end{aligned}$$

denotes the Frobenius (matrix) norm of $\sigma$. In particular, (H3) implies that there exists a positive constant $\sigma _{*}\ge 0$ such that

$$\begin{aligned} ||\sigma (x,t)||^{2} \le \sigma _{*}^{2}\quad \text {for all } x\in \mathcal {X}, t\ge 0. \end{aligned}$$

In what follows, it will be convenient to measure the intensity of the noise affecting (SMD) via $\sigma _{*}$; of course, when $\sigma _{*} = 0$, we recover the noiseless, deterministic dynamics (MD).

## Deterministic Analysis

To establish a reference standard, we first focus on the deterministic regime of (MD), i.e., when $M(t)\equiv 0$ in (SMD).

### Global Existence

We begin with a basic well-posedness result of (MD).

#### Proposition 3.1

Under Hypotheses (H1) and (H2), the dynamical system (MD) admits a unique solution from every initial condition $(s,y)\in \mathbb {R}_{+}\times \mathcal {Y}$.

#### Proof

Let $A(t,y) := -\lambda (t) v(Q(\eta (t)y))$ for all $t\in \mathbb {R}_+$, $y\in \mathcal {Y}$. Clearly, *A*(*t*, *y*) is jointly continuous in *t* and *y*. Moreover, by (H2), $\lambda (t)$ has bounded first derivative and $\eta (t)$ is nonincreasing, so both $\lambda (t)$ and $\eta (t)$ are Lipschitz continuous. Finally, by (H1), *v* is *L*-Lipschitz continuous, implying in turn that

$$\begin{aligned} ||A(t,y_{1})-A(t,y_{2})||_{*} \le \frac{L\eta (t)\lambda (t)}{\alpha } ||y_{1}-y_{2}||_{*} \quad \text {for all } y_{1},y_{2}\in \mathcal {Y}, \end{aligned}$$

where $\alpha$ is the strong convexity constant of *h*, and we used Proposition 2.1 to estimate the Lipschitz constant of *Q*. This shows that *A*(*t*, *y*) is Lipschitz in *y* for all *t*, so existence and uniqueness of local solutions follows from the Picard–Lindelöf theorem. Hypothesis (H2) further guarantees that the Lipschitz constant of $A(t,\cdot )$ can be chosen uniformly in *t*, so these solutions can be extended for all $t\ge 0$. $\square$

Let $\mathbb {T}:=\{(t,s)\vert 0\le s\le t\le \infty \}$. Based on the above, we may define a *nonautonomous semiflow* $Y:\mathbb {T}\times \mathcal {Y}\rightarrow \mathcal {Y}$ satisfying (i) $Y(s,s,y)=y$ for all $s\ge 0$, (ii) $\frac{\partial Y(t,s,y)}{\partial t}=A(t,Y(t,s,y))$ for all $(t,s,y)\in \mathbb {T}\times \mathcal {Y}$, and (iii) $Y(t,s,Y(s,r,y))=Y(t,r,y)$ for $t\ge s\ge r\ge 0$. Since the dynamics will usually be started from an initial condition $(0,y)\in \mathbb {R}_{+}\times \mathcal {Y}$, we will simplify the notation by writing $\phi (t,y)=Y(t,0,y)$ for all $(t,y)\in \mathbb {R}_{+}\times \mathcal {Y}$. The resulting trajectory in the primal space is denoted by $\xi (t,y)=Q(\eta (t)\phi (t,y))$. Note that, if $\lambda (t)$ and $\eta (t)$ are constant functions, then the mapping $\phi (t,y)$ is the (autonomous) *semiflow* of the dynamics (MD).

### Convergence Properties and Performance

Now, to analyze the convergence of (MD), we will consider two “gap functions” quantifying the distance between the primal trajectory, and the solution set of (MVI):

- In the general case, we will focus on the *dual gap* function [25]:
  $$\begin{aligned} g(x):= \max _{x'\in \mathcal {X}}\langle v(x'),x - x'\rangle . \end{aligned}$$
  By (H1) and the compactness of $\mathcal {X}$, it follows that *g*(*x*) is continuous, nonnegative and convex; moreover, we have $g(x) = 0$ if and only if *x* is a solution of ${{\mathrm{VI}}}(\mathcal {X},v)$ [7, Proposition 3.1].
- For the saddle-point problem Ex. 2.2, we instead look at the *Nikaido–Isoda gap function* [26]:
  3 $$\begin{aligned} G(p^{1},p^{2}):= \max _{x^{2}\in \mathcal {X}^{2}}U(p^{1},x^{2}) - \min _{x^{1}\in \mathcal {X}^{1}} U(x^{1},p^{2}). \end{aligned}$$

Since *U* is convex-concave, it is immediate that $G(p^{1},p^{2})\ge g(p^{1},p^{2})$, where the operator involved in the definition of the dual gap function is given by the saddle-point operator (1). However, it is still true that $G(p^{1},p^{2})=0$ if and only if the pair $(p^{1},p^{2})$ is a saddle-point. Since both gap functions vanish only at solutions of (MVI), we will prove trajectory convergence by monitoring the decrease of the relevant gap over time. This is achieved by introducing the so-called *Fenchel coupling* [14], an auxiliary energy function, defined as

$$\begin{aligned} F(x,y):= h(x) + h^{*}(y) - \langle y,x\rangle \quad \text {for all } x\in \mathcal {X}, y\in \mathcal {Y}. \end{aligned}$$

Some key properties of *F* are summarized in the following proposition:

#### Proposition 3.2

([14]) Let *h* be a distance-generating function on $\mathcal {X}$. Then:

- $$F(x,y)\ge \frac{\alpha }{2} ||Q(y)-x||^{2}$$ for all $$x\in \mathcal {X}$$, $$y\in \mathcal {Y}$$.
- Viewed as a function of *y*, *F*(*x*, *y*) is convex, differentiable, and its gradient is given by
  $$\begin{aligned} \nabla _{y} F(x,y) = Q(y) - x. \end{aligned}$$
- For all $x\in \mathcal {X}$ and all $y,y'\in \mathcal {Y}$, we have
  $$\begin{aligned} F(x,y') \le F(x,y) + \langle y'-y,Q(y)-x\rangle + \frac{1}{2\alpha } ||y' - y||_{*}^{2}. \end{aligned}$$

In the sequel, if there is no danger of confusion, we will use the more concise notation $x(t)=\xi (t,y)$ and $y(t)=\phi (t,y)$, for the unique solution to (MD) with initial condition $(0,y)\in \mathbb {R}_{+}\times \mathcal {Y}$. Consider the averaged trajectory

4 $$\begin{aligned} \bar{x}(t):= \frac{\int _{0}^{t} \lambda (s) x(s) \,\mathrm{d}s}{\int _{0}^{t} \lambda (s) \,\mathrm{d}s}= \frac{1}{S(t)} \int _{0}^{t} \lambda (s) x(s) \,\mathrm{d}s, \end{aligned}$$

where $S(t):= \int _{0}^{t} \lambda (s) \,\mathrm{d}s.$ We then have the following convergence guarantee:

#### Proposition 3.3

Suppose that (MD) is initialized at $(s,y)=(0,0)$, with resulting trajectories $y(t)=\phi (t,0)$ and $x(t)=\xi (t,0)$. Then:

5 $$\begin{aligned} g(\bar{x}(t)) \le \frac{\mathcal {D}(h;\mathcal {X})}{\eta (t)S(t)}, \end{aligned}$$

where $\bar{x}(t)$ is the averaged trajectory constructed in (4), and

$$\begin{aligned} \mathcal {D}(h;\mathcal {X}):= \max _{x,x'\in \mathcal {X}} \{ h(x') - h(x) \}= \max h - \min h. \end{aligned}$$

In particular, if (MVI) is associated with a convex-concave saddle-point problem as in Example 2.2, we have the guarantee:

6 $$\begin{aligned} G(\bar{x}(t)) \le \frac{\mathcal {D}(h_{1};\mathcal {X}^{1})+\mathcal {D}(h_{2};\mathcal {X}^{2})}{\eta (t)S(t)}. \end{aligned}$$

In both cases, whenever $\lim _{t\rightarrow \infty } \eta (t) S(t) = \infty$, $\bar{x}(t)$ converges to the solution set of ${{\mathrm{VI}}}(\mathcal {X},v)$.

#### Proof

Given some $p\in \mathcal {X}$, let $H_{p}(t):= \frac{1}{\eta (t)}F(p,\eta (t)y(t))$. Then, with Proposition 3.2, the fundamental theorem of calculus yields

$$\begin{aligned} H_{p}(t) - H_{p}(0) = -\int _{0}^{t}\lambda (s) \langle v(x(s)),x(s)-p\rangle \,\mathrm{d}s - \int _{0}^{t} \frac{\dot{\eta }(s)}{\eta (s)^{2}}[h(p)-h(x(s))] \,\mathrm{d}s, \end{aligned}$$

and, after rearranging, we obtain

7 $$\begin{aligned} \int _{0}^{t}\lambda (s)\langle v(x(s)),x(s)-p\rangle \,\mathrm{d}s&=H_{p}(0)-H_{p}(t)-\int _{0}^{t}\frac{\dot{\eta }(s)}{\eta (s)^{2}}[h(p)-h(x(s))]\,\mathrm{d}s\nonumber \\&\le H_{p}(0)+\mathcal {D}(h;\mathcal {X})\left( \frac{1}{\eta (t)}-\frac{1}{\eta (0)}\right) . \end{aligned}$$

Now, let $x_{c}:={{\mathrm{argmin}}}\{h(x):x\in \mathcal {X}\}$ denote the “prox-center” of $\mathcal {X}$. Since $\eta (0)>0$ and $y(0)=0$ by assumption, we readily get

8 $$\begin{aligned} H_{p}(0) = \frac{F(p,0)}{\eta (0)} =\frac{h(p) + h^{*}(0) - \langle 0,p\rangle }{\eta (0)} =\frac{h(p)-h(x_{c})}{\eta (0)} \le \frac{\mathcal {D}(h;\mathcal {X})}{\eta (0)}. \end{aligned}$$

From the monotonicity of *v*, we further deduce that

9 $$\begin{aligned} g(\bar{x}(t)) \le \frac{1}{S(t)} \max _{p\in \mathcal {X}} \int _{0}^{t} \lambda (s)\langle v(x(s)),x(s)-p\rangle \,\mathrm{d}s. \end{aligned}$$

Thus, substituting (8) in (7), maximizing over $p\in \mathcal {X}$ and plugging the result into (9) gives (5).

Suppose now that (MVI) is associated to a convex-concave saddle-point problem, as in Ex. 2.2. In this case, we can replicate the above analysis for each component $x^{i}(t)$, $i=1,2$, of *x*(*t*) to obtain the basic bounds

$$\begin{aligned} \int _{0}^{t}\lambda (s)\langle \nabla _{x^{1}}U(x(s)),x^{1}(s)-p^{1}\rangle \,\mathrm{d}s&\le \frac{\mathcal {D}(h_{1};\mathcal {X}^{1})}{\eta (t)},\\ \int _{0}^{t}\lambda (s)\langle -\nabla _{x^{2}}U(x(s)),x^{2}(s)-p^{2}\rangle \,\mathrm{d}s&\le \frac{\mathcal {D}(h_{2};\mathcal {X}^{2})}{\eta (t)}. \end{aligned}$$

Using the fact that *U* is convex-concave, this leads to the value-based bounds

$$\begin{aligned} \int _{0}^{t}\lambda (s)[U(x(s))-U(p^{1},x^{2}(s))] \,\mathrm{d}s&\le \frac{\mathcal {D}(h_{1};\mathcal {X}^{1})}{\eta (t)},\\ \int _{0}^{t}\lambda (s)[U(x^{1}(s),p^{2})-U(x(s))] \,\mathrm{d}s&\le \frac{\mathcal {D}(h_{2};\mathcal {X}^{2})}{\eta (t)}. \end{aligned}$$

Summing these inequalities, dividing by *S*(*t*), and using Jensen’s inequality gives

$$\begin{aligned} U(\bar{x}^{1}(t),p^{2})-U(p^{1},\bar{x}^{2}(t))\le \frac{\mathcal {D}(h_{1};\mathcal {X}^{1})+\mathcal {D}(h_{2};\mathcal {X}^{2})}{\eta (t)S(t)} \end{aligned}$$

The bound (6) then follows by taking the supremum over $p^{1}$ and $p^{2}$, and using the definition of the Nikaido– Isoda gap function. $\square$

The gap-based analysis of Proposition 3.3 can be refined further in the case of *strongly* monotone VIs.

#### Proposition 3.4

Let $x_{*}$ denote the (necessarily unique) solution of a $\gamma$-strongly monotone ${{\mathrm{VI}}}(\mathcal {X},v)$. Then, with the same assumptions as in Proposition 3.3, we have

10 $$\begin{aligned} ||\bar{x}(t) - x_{*}||^{2} \le \frac{\mathcal {D}(h;\mathcal {X})}{\gamma } \frac{1}{\eta (t)S(t)}. \end{aligned}$$

In particular, $\bar{x}(t)$ converges to $x_{*}$ whenever $\lim _{t\rightarrow \infty } \eta (t) S(t) = \infty$.

#### Proof

By Jensen’s inequality, the strong monotonicity of *v* and the assumption that $x_{*}$ solves ${{\mathrm{VI}}}(\mathcal {X},v)$, we have:

$$\begin{aligned} \begin{array}{llr} \gamma ||\bar{x}(t) - x_{*}||^{2} &{}\le \frac{\gamma }{S(t)} \int _{0}^{t} \lambda (s) ||*||{x(s) - x_{*}}^{2} \,\mathrm{d}s &{}\quad {\mathrm{(Jensen)}}\\ &{}\le \frac{1}{S(t)} \int _{0}^{t} \lambda (s) \langle v(x(s)) - v(x_{*}), x(s) - x_{*}\rangle \,\mathrm{d}s &{}\quad (\gamma {\mathrm{-monotonicity}})\\ &{}\le \frac{1}{S(t)} \int _{0}^{t} \lambda (s) \langle v(x(s)), x(s) - x_{*}\rangle \,\mathrm{d}s &{}\quad ({\mathrm{optimality}}~{\mathrm{of }}\, x_{*})\\ &{}\le \frac{\mathcal {D}(h;\mathcal {X})}{\eta (t)S(t)},&{} \end{array} \end{aligned}$$

where the last inequality follows as in the proof of Proposition 3.3. The bound (10) is then obtained by dividing both sides by $\gamma$. $\square$

The two results above are in the spirit of classical ergodic convergence results for monotone VIs [13, 27, 28]. In particular, taking $\eta (t)=\sqrt{L/(2\alpha )}$ and $\lambda (t) = 1/(2\sqrt{t})$ gives the upper bound $g(\bar{x}(t))\le \mathcal {D}(h;\mathcal {X}) \sqrt{L/(\alpha t)}$, which is of the same order as the $\mathcal {O}(1/\sqrt{t})$ guarantees obtained in the references above. However, the bound (9) does not have a term which is antagonistic to $\eta (t)$ or $\lambda (t)$, so, if (MD) is run with constant $\lambda$ and $\eta$, we get an $\mathcal {O}(1/t)$ bound for $g(\bar{x}(t))$ (and/or $||\bar{x}(t) - x_{*}||$ in the case of strongly monotone VIs).^7^ This suggests an important gap between continuous and discrete time; for a similar phenomenon in the context of online convex optimization, see the regret minimization analysis of [29].

We close this section with a (nonergodic) trajectory convergence result for strictly monotone problems. For any path $X(\cdot ):\mathbb {R}_{+}\rightarrow \mathcal {X}$, call the *limit set*

$$\begin{aligned} \mathcal {L}\{X(\cdot )\}:=\bigcap _{t\ge 0}{{\mathrm{cl}}}[X([t,\infty )]. \end{aligned}$$

#### Proposition 3.5

Let $x_{*}$ denote the (necessarily unique) solution of a $\gamma$-strongly monotone ${{\mathrm{VI}}}(\mathcal {X},v)$. Suppose that Hypotheses (H1) and (H2) hold, and the parameters $\lambda$ and $\eta$ of (MD) satisfy

$$\begin{aligned} \textstyle \inf _{t} \lambda (t)> 0 \quad \text {and} \quad \inf _{t} \eta (t) > 0. \end{aligned}$$

Then, $\lim _{t\rightarrow \infty } \xi (t,y) = x_{*}$, for any $y\in \mathcal {Y}$.

#### Proof

Let $x(t):=\xi (t,y)$ for $t\ge 0$, and assume that $\hat{x}\in \mathcal {L}\{x(\cdot )\}$, but $\hat{x}\ne x_{*}$. Then, by assumption, there exists an open neighborhood *O* of $\hat{x}$ and a positive constant $a>0$ such that

$$\begin{aligned} \langle v(x),x-x_{*}\rangle \ge a \quad \text {for all } x\in O. \end{aligned}$$

Furthermore, since $\hat{x}$ is an accumulation point of *x*(*t*), there exists an increasing sequence $(t_{k})_{k\in \mathbb {N}}$ such that $t_{k}\uparrow \infty$ and $x(t_{k}) \rightarrow \hat{x}$ as $k\rightarrow \infty$. Thus, relabeling indices if necessary, we may assume without loss of generality that $x(t_{k})\in O$ for all $k\in \mathbb {N}$. Now, for all $\varepsilon >0$, we have

$$\begin{aligned} ||x(t_{k}+\varepsilon )-x(t_{k})||&=||Q(Y(t_{k}+\varepsilon ))-Q(Y(t_{k}))||\\&\le \frac{1}{\alpha }||Y(t_{k}+\varepsilon )-Y(t_{k})||_{*}\\&\le \frac{1}{\alpha }\int _{t_{k}}^{t_{k}+\varepsilon }\lambda (s)||v(x(s))||_{*}\,\mathrm{d}s\\&\le \frac{1}{\alpha }\max _{x\in \mathcal {X}}||v(x)||_{*}\int _{t_{k}}^{t_{k}+\varepsilon } \lambda (s)\,\mathrm{d}s \\&\le \frac{\varepsilon \bar{\lambda }}{\alpha }\max _{x\in \mathcal {X}}||v(x)||_{*}, \end{aligned}$$

where $\bar{\lambda }:= \lambda (0)$ denotes the maximum value of $\lambda (t)$. As this bound does not depend on *k*, we can choose $\varepsilon >0$ small enough so that $x(t_{k}+s)\in O$ for all $s\in [0,\varepsilon ]$ and all $k\in \mathbb {N}$. Thus, letting $H(t) := \eta (t)^{-1} F(x_{*},\eta (t) y(t))$, and using (7), we obtain

$$\begin{aligned} H(t_{n}) - H(t_{0})&= -\sum _{k=1}^{n} \int _{t_{k-1}}^{t_{k}} \lambda (s) \langle v(x(s)),x(s) - x_{*}\rangle \,\mathrm{d}s +\mathcal {D}(h;\mathcal {X})\left( \frac{1}{\eta (t_{n})}-\frac{1}{\eta (t_{0})}\right) \\&\le -a\underline{\lambda } \sum _{k=1}^{n}(t_{k}-t_{k-1})+\mathcal {D}(h;\mathcal {X}) \left( \frac{1}{\eta (t_{n})}-\frac{1}{\eta (t_{0})}\right) \\&=-a\varepsilon \underline{\lambda }n+\mathcal {D}(h;\mathcal {X}) \left( \frac{1}{\eta (t_{n})}-\frac{1}{\eta (t_{0})}\right) , \end{aligned}$$

where we have set $\underline{\lambda }:= \inf _{t}\lambda (t) > 0$. Given that $\inf _{t}\eta (t) > 0$, the above implies that $\lim _{n\rightarrow \infty } H(t_{n}) = -\infty$, contradicting the fact that $F(x_{*},y)\ge 0$ for all $y\in \mathcal {Y}$. This implies that $\hat{x}=x_{*}$; by compactness, $\mathcal {L}\{x(\cdot )\}\ne \varnothing$, so our claim follows. $\square$

## Analysis of the Stochastic Dynamics

### Global Existence

In this section, we turn to the stochastic system (SMD). As in the noise-free analysis of the previous section, we begin with a well-posedness result, stated for simplicity for deterministic initial conditions.

#### Proposition 4.1

Fix an initial condition $(s,y)\in \mathbb {R}_{+}\times \mathcal {Y}$. Then, under Hypotheses (H1)–(H3), and up to a ${{\mathrm{\mathbb {P}}}}$-null set, the stochastic dynamics (SMD) admit a unique strong solution $(Y(t))_{t\ge s}$ such that $Y(s) = y$.

#### Proof

Let $B(t,y):=- \lambda (t) \sigma (Q(\eta (t)y),t)$ so (SMD) can be written as

11 $$\begin{aligned} dY(t)= A(t,Y(t))\,\mathrm{d}t+B(t,Y(t)) \,\mathrm{d}W(t), \end{aligned}$$

with *A*(*t*, *y*) defined as in the proof of Proposition 3.1. By (H2) and (H3), *B*(*t*, *y*) inherits the boundedness and regularity properties of $\sigma$; in particular, Hypotheses (H2) and (H3), together with Proposition 2.1c), imply that *B*(*t*, *y*) is uniformly Lipschitz in *y*. Under Hypotheses (H1) and (H3), *A*(*t*, *y*) is also uniformly Lipschitz in *y* (cf. the proof of Proposition 3.1). Our claim then follows by standard results in the well posedness of stochastic differential equations [30, Theorem 3.4]. $\square$

We denote by *Y*(*t*, *s*, *y*) the unique strong solution of the Itô stochastic differential equation (11), with initial condition $(s,y)\in \mathbb {R}_{+}\times \mathcal {Y}$. As in the deterministic case, we are mostly interested in the process starting from the initial condition (0, *y*), in which case we abuse the notation by writing $Y(t,y)=Y(t,0,y)$. The corresponding primal trajectories are generated by applying the mirror map *Q* to the dual trajectories, so $X(t,y)=Q(\eta (t)Y(t,y))$, for all $(t,y)\in \mathbb {R}_{+}\times \mathcal {Y}$. If there is no danger of confusion, we will consistently suppress the dependence on the initial position $y\in \mathcal {Y}$ in both random processes. Clearly, if $\lambda (t)$ and $\eta (t)$ are constant function, the solutions of (SMD) are time-autonomous.

We now give a brief overview of the results we obtain in this section. First, in Sect. 4.2, we use the theory of *asymptotic pseudo-trajectories* (APTs), developed by Benaïm and Hirsch [31], to establish almost sure trajectory convergence of (SMD) to the solution of ${{\mathrm{VI}}}(\mathcal {X},v)$, provided that *v* is strictly monotone and the oracle noise in (SMD) is vanishing at a rather slow, logarithmic rate. This strong convergence result relies heavily on the shadowing property of the dual trajectory, and its deterministic counterpart $\phi (t,y)$ (see Sect.  4.2). On the other hand, if the driving noise process is persistent, we cannot expect the primal trajectory *X*(*t*) to converge—some averaging has to be done in this case. Thus, following a long tradition on ergodic convergence for mirror descent, we investigate in Sect. 4.3 the asymptotics of a weighted time-average of *X*(*t*). Finally, we complement our ergodic convergence results with a large deviation principle showing that the ergodic average of *X*(*t*) is exponentially concentrated around its mean (Sect. 4.4).

### The Small Noise Limit

We begin with the case, where the oracle noise in (SMD) satisfies the asymptotic decay condition $||\sigma (x,t)|| \le \beta (t)$ for some nonincreasing function $\beta :\mathbb {R}_+\rightarrow \mathbb {R}_+$ such that

$$\begin{aligned} {\mathrm{(H4)}}\qquad \int _{0}^{\infty } \exp \left( -\frac{c}{\beta ^{2}(t)}\right) \,\mathrm{d}t < \infty \quad \text {for all } c>0. \end{aligned}$$

For instance, this condition is trivially satisfied if $\sigma (x,t)$ vanishes at a logarithmic rate, i.e., $\beta (t) = o(1/\sqrt{\log (t)})$. For technical reasons, we will also need the additional “Fenchel reciprocity” condition

$$\begin{aligned} \mathrm{(H5)}\qquad F(p,y_{n})\rightarrow 0 \quad \text {whenever} \quad Q(y_{n})\rightarrow p. \end{aligned}$$

Under the decay rate requirement (H4), and working for simplicity with constant $\eta (t) = \lambda (t) = 1$, the results of [31, Proposition 4.1] imply that any strong solution *Y*(*t*) of (SMD) is an (APT) of the deterministic dynamics (MD) in the following sense:

#### Definition 4.1

Assume that $\eta (t) = \lambda (t) = 1$, for all $t\ge 0$. Let $\phi :\mathbb {R}_+\times \mathcal {Y}\rightarrow \mathcal {Y}$, $(t,y)\mapsto \phi (t,y)$, denote the semiflow induced by (MD) on $\mathcal {Y}$. A continuous curve $Y:\mathbb {R}_+\rightarrow \mathcal {Y}$ is said to be an *asymptotic pseudo-trajectory* (APT) of (MD), if

APT $$\begin{aligned} \lim _{t\rightarrow \infty } \sup _{0\le s\le T} ||Y(t+s) - \phi (s,Y(t))||_{*} = 0 \quad \text {for all }T>0. \end{aligned}$$

In words, Definition 4.1 states that an APT of (MD) tracks the solutions of (MD) to arbitrary accuracy over arbitrarily long time windows. Thanks to this property, we are able to establish the following global convergence theorem for (SMD) with vanishing oracle noise:

#### Theorem 4.1

Assume that *v* is strictly monotone, and let $x_{*}$ denote the (necessarily unique) solution of ${{\mathrm{VI}}}(\mathcal {X},v)$. If Hypotheses (H1)–(H5) hold, and (SMD) is run with $\lambda (t) = \eta (t) = 1$, we have

$$\begin{aligned} \mathbb {P}\left( \lim _{t\rightarrow \infty } ||X(t,y) - x_{*}|| = 0\right) =1 \quad \forall y\in \mathcal {Y}. \end{aligned}$$

The proof of Theorem 4.1 requires some auxiliary results, which we provide below. We begin with a strong recurrence result for neighborhoods of the (unique) solution $x_{*}$ of ${{\mathrm{VI}}}(\mathcal {X},v)$ under (MD):

#### Lemma 4.1

With assumptions as in Theorem 4.1, let $\mathcal {O}$ be an open neighborhood of $x_{*}$ in $\mathcal {X}$ and let $\xi (t,y)=Q(\eta (t)\phi (t,y))$. Define the stopping time

$$\begin{aligned} t_{\mathcal {O}}(y) := \inf \{t\ge 0:\xi (t,y)\in \mathcal {O}\}. \end{aligned}$$

Then, $t_{\mathcal {O}}(y)<\infty$ for all $y\in \mathcal {Y}$.

#### Proof

Fix the initialization $y\in \mathcal {Y}$ of (MD), and let $y(t):= \phi (t,y),\;x(t):=Q(\phi (t,y))$ denote the induced solutions of (MD), and let $H(t) := F(x_{*},y(t))$. Then, by Proposition 3.2, and the chain rule applied to (MD), we get

$$\begin{aligned} H(t) = H(0) - \int _{0}^{t} \langle v(x(s)),x(s)-x_{*}\rangle \,\mathrm{d}s. \end{aligned}$$

Since *v* is strictly monotone and $x_{*}$ solves ${{\mathrm{VI}}}(\mathcal {X},v)$, there exists some $a\equiv a_{\mathcal {O}} > 0$ such that

$$\begin{aligned} \langle v(x),x-x_{*}\rangle \ge a \quad \text {for all } x\in \mathcal {X}\setminus \mathcal {O}. \end{aligned}$$

Hence, if $t_{\mathcal {O}}(y) = \infty$, we would have

$$\begin{aligned} H(t) \le H(0) - a t \quad \text {for all} t\ge 0, \end{aligned}$$

implying in turn that $\lim _{t\rightarrow \infty } H(t) = -\infty$. This contradicts the fact that $H(t)\ge 0$, so we conclude that $t_{\mathcal {O}}(y) < \infty$. $\square$

Next, we extend this result to the stochastic regime:

#### Lemma 4.2

With assumptions as in Theorem 4.1, let $\mathcal {O}$ be an open neighborhood of $x_{*}$ in $\mathcal {X}$ and define the stopping time

$$\begin{aligned} \tau _{\mathcal {O}}(y):=\inf \{t\ge 0: X(t,y)\in \mathcal {O}\}, \end{aligned}$$

Then, $\tau _{\mathcal {O}}(y)$ is almost surely finite for all $y\in \mathcal {Y}$.

#### Proof

Suppose there exists some initial condition $y_{0}\in \mathcal {Y}$, such that $\mathbb {P}\left( \tau _{\mathcal {O}}(y_{0})=\infty \right) >0$. Then, there exists a measurable set $\varOmega _{0}\subseteq \varOmega$, with $\mathbb {P}\left( \varOmega _{0}\right) >0$, and such that $\tau _{\mathcal {O}}(\omega ,y_{0})=\infty$ for all $\omega \in \varOmega _{0}$. Now, define $H(t):=F(x_{*},Y(t,y_{0}))$ and set $X(t)=X(t,y_{0})$. By the weak Itô lemma (33) proven in Sect. 5, we get

$$\begin{aligned} H(t) - H(0) \le -\int _{0}^{t} \langle v(X(s)),X(s) - x_{*}\rangle \,\mathrm{d}s + \frac{1}{2\alpha } \int _{0}^{t} ||\sigma (X(s),s)||^{2}\,\mathrm{d}s + I_{x_{*}}(t) \end{aligned}$$

where $I_{x_{*}}(t):= \int _{0}^{t} \langle X(s) - x_{*},\sigma (X(s))\cdot \,\mathrm{d}W(s)\rangle$ is a continuous local martingale. Since *v* is strictly monotone, the same reasoning as in the proof of Lemma 4.1 yields

$$\begin{aligned} H(t) \le H(0) - at + I_{x_{*}}(t) + \frac{\sigma _{*}^{2}}{2\alpha } \end{aligned}$$

for some $a \equiv a_{\mathcal {O}} > 0$ and for all $t\in [0,\tau _{\mathcal {O}}(y))$. Furthermore, by an argument based on the law of the iterated logarithm and the Dambis–Dubins–Schwarz time-change theorem for martingales as in the proof of Theorem 4.2, we get

$$\begin{aligned} I_{x_{*}}(t)/t \rightarrow 0 \text { almost surely as } t\rightarrow \infty . \end{aligned}$$

Combining this with the estimate for *H*(*t*) above, we get $\lim _{t\rightarrow \infty } H(t) = -\infty$ for ${{\mathrm{\mathbb {P}}}}$-almost all $\omega \in \varOmega _{0}$. This contradicts the fact that $H(t)\ge 0$, and our claim follows. $\square$

The above result shows that the primal process *X*(*t*) hits any neighborhood of $x_{*}$ in finite time (a.s.). Thanks to this important recurrence property, we are finally in a position to prove Theorem 4.1:

#### Proof of Theorem 4.1

Fix some $\varepsilon >0$, and let $N_{\varepsilon }:=\{x=Q(y):F(x_{*},y)<\varepsilon \}$. Let $y\in \mathcal {Y}$ be arbitrary. We first claim, that there exists a deterministic time $T\equiv T(\varepsilon )$ such that $F(x_{*},\phi (T,y))\le \max \{\varepsilon ,F(x_{*},y)+\varepsilon \}$. Indeed, consider the hitting time

$$\begin{aligned} t_{\varepsilon }(y):=\inf \{t\ge 0:x(t)\in N_{\varepsilon }\}, \end{aligned}$$

where $x(t) :=Q(\phi (t,y))$. By Hypothesis (H5), $N_{\varepsilon }$ contains a neighborhood of $x_{*}$; hence, by Lemma 4.1, we have $t_{\varepsilon }(y) < \infty$. Moreover, observe that

12 $$\begin{aligned} \frac{\mathrm{d}}{\mathrm{d}t} F(x_{*},\phi (t,y)) =-\langle v(x(t)),x(t) - x_{*}\rangle \le 0 \quad \text {for all } y\in \mathcal {Y}. \end{aligned}$$

The strict monotonicity of *v* and the fact that $x_{*}$ solves (MVI) imply that there exists a positive constant $\kappa \equiv \kappa _{\varepsilon } >0$ such that $\langle v(x),x-x_{*}\rangle \ge \kappa$ for all $x\in \mathcal {X}\setminus N_{\varepsilon }$. Hence, combining this with (12), we readily see that

$$\begin{aligned} F(x_{*},\phi (t,y)) - F(x_{*},y) \le -\kappa t \quad \text {for all } t\in [0,t_{\varepsilon }(y)). \end{aligned}$$

Now, set $T = \varepsilon /\kappa$. If $T<t_{\varepsilon }(y)$, we immediately conclude that

$$\begin{aligned} F(x_{*},\phi (T,y)) - F(x_{*},y) \le -\varepsilon . \end{aligned}$$

Otherwise, if $T \ge t_{\varepsilon }(y)$, we again use the descent property (12) to get

$$\begin{aligned} F(x_{*},\phi (T,y)) \le F(x_{*},\phi (t_{\varepsilon }(y),y)) \le \varepsilon . \end{aligned}$$

In both cases we have $F(x_{*},\phi (T,y)) \le \max \{\varepsilon ,F(x_{*},y)-\varepsilon \}$, as claimed.

To proceed, pick $\delta \equiv \delta _{\varepsilon }>0$ such that

13 $$\begin{aligned} \delta _{\varepsilon }{{\mathrm{diam}}}(\mathcal {X})+\frac{\delta ^{2}_{\varepsilon }}{2\alpha }<\varepsilon , \end{aligned}$$

where ${{\mathrm{diam}}}(\mathcal {X}):= \max \{||x'-x||_{2}:x,x'\in \mathcal {X}\}$ denotes the Euclidean diameter of $\mathcal {X}$. By Proposition 4.1 of [31], the strong solution *Y* of (11) (viewed as a stochastic flow) is an APT of the deterministic semiflow $\phi$ with probability 1. Hence, we can choose an (a.s.) finite random time $\theta _{\varepsilon }$ such that $\sup _{s\in [0,T]}||Y(t+s)-\phi (s,Y(t))||_{*}\le \delta _{\varepsilon }$ for all $t\ge \theta _{\varepsilon }$. Combining this with item (c) of Proposition 3.2, we then get

$$\begin{aligned} F(x_{*},Y(t+s,y))&\le F(x_{*},\phi (s,Y(t,y)))\\&\quad +\langle Y(t+s,y)-\phi (s,Y(t,y)),Q(\phi (s,Y(t,y)))-x_{*}\rangle \\&\quad +\frac{1}{2\alpha }||Y(t+s,y)-\phi (s,Y(t,y))||_{*}^{2}\\&\le F(x_{*},\phi (s,Y(t,y)))+\delta _{\varepsilon }{{\mathrm{diam}}}(\mathcal {X}) +\frac{\delta _{\varepsilon }^{2}}{2\alpha }\\&\le F(x_{*},\phi (s,Y(t,y)))+\varepsilon , \end{aligned}$$

where the last inequality follows from the estimate (13).

Now, choose a random time $T_{0}\ge \max \{\theta _{\varepsilon }(y),t_{\varepsilon }(y)\}$ and $T=\varepsilon /\kappa$ as above. Then, by definition, we have $F(x_{*},Y(T_{0},y))\le 2\varepsilon$ with probability 1. Hence, for all $s\in [0,T]$, we get

$$\begin{aligned} F(x_{*},Y(T_{0}+s,y)) \le F(x_{*},\phi (s,Y(T_{0},y))) + \varepsilon \le F(x_{*},Y(T_{0},y)) + \varepsilon \le 3\varepsilon . \end{aligned}$$

Since $F(x_{*},\phi (T,Y(T_{0},y))) \le \max \{\varepsilon ,F(x_{*},Y(T_{0},y)) - \varepsilon \}\le \varepsilon$, we also get

$$\begin{aligned} F(x_{*},Y(T_{0}+T+s,y))&\le F(x_{*},\phi (s,Y(T_{0}+T,y))) + \varepsilon \\&\le F(x_{*},Y(T_{0}+T,y)) + \varepsilon \\&\le 3\varepsilon , \end{aligned}$$

and hence

$$\begin{aligned} F(x_{*},Y(T_{0}+s,y))\le 3\varepsilon \quad \text {for all } s\in [T,2T]. \end{aligned}$$

Using this as the basis for an induction argument, we readily get

$$\begin{aligned} F(x_{*},Y(T_{0}+s,y)) \le 3\varepsilon \quad \text {for all } s\in [nT,(n+1)T], \end{aligned}$$

with probability 1. Since $\varepsilon$ was arbitrary, we obtain $F(x_{*},Y(t,y))\rightarrow 0$, implying in turn that $X(t)\rightarrow x_{*}$ (a.s.) by Proposition 3.2. $\square$

### Ergodic Convergence

We now proceed with an ergodic convergence result, in the spirit of Proposition 3.3. The results presented in this section are derived under the assumption that (SMD) is started with the initial conditions $(s,y)=(0,0)$. This is only done to make the presentation clearer; see Remark 4.1.

Set $S(t):= \int _{0}^{t} \lambda (s) \,\mathrm{d}s$, $L(t):= \sqrt{\int _{0}^{t} \lambda ^{2}(s) \,\mathrm{d}s}$, and let

$$\begin{aligned} \bar{X}(t):=\frac{1}{S(t)}\int _{0}^{t}\lambda (s)X(s)\,\mathrm{d}s, \end{aligned}$$

denote the “ergodic average” of $X(t)=X(t,0,0)$.

#### Theorem 4.2

Under Hypotheses (H1)–(H3), we have:

14 $$\begin{aligned} g(\bar{X}(t)) = \mathcal {O}\left( \frac{1}{\eta (t) S(t)}\right) + \mathcal {O}\left( \frac{\int _{0}^{t} \lambda ^{2}(s) \eta (s)\,\mathrm{d}s}{S(t)}\right) + \mathcal {O}\left( \frac{L(t) \sqrt{\log \log L(t)}}{S(t)}\right) , \end{aligned}$$

with probability 1. In particular, $\bar{X}(t)$ converges (a.s.) to the solution set of ${{\mathrm{VI}}}(\mathcal {X},v)$ provided that a) $\lim _{t\rightarrow \infty } \eta (t) S(t) = \infty$; and b) $\lim _{t\rightarrow \infty } \eta (t) \lambda (t) = 0$.

Before discussing the proof of Theorem 4.2, it is worth noting the interplay between the two variable weight parameters, $\lambda (t)$ and $\eta (t)$. In particular, if (SMD) is run with weight sequences of the form $1/t^{q}$ for some $q>0$, we obtain:

#### Corollary 4.1

Suppose that (SMD) is run with $\lambda (t) = (1+t)^{-a}$ and $\eta (t) = (1+t)^{-b}$ for some $a,b\in [0,1]$. Then, with assumptions as in Theorem 4.2, we have:

15 $$\begin{aligned} g(\bar{X}(t)) = \tilde{\mathcal {O}}\left( t^{a+b-1}\right) + \tilde{\mathcal {O}}\left( t^{-a-b}\right) + \tilde{\mathcal {O}}\left( t^{-1/2}\right) . \end{aligned}$$

In the above, the $\tilde{\mathcal {O}}(\cdot )$ notation signifies “$\mathcal {O}(\cdot )$ up to logarithmic factors”.^8^ Up to such factors, (15) is optimized when $a+b=1/2$; if these factors are to be considered, any choice with $a+b=1/2$ and $b>0$ gives the same rate of convergence, indicating that the role of the post-multiplication factor $\eta (t)$ is crucial to finetune the convergence rate of (SMD). We find this observation particularly appealing, as it is reminiscent of Nesterov’s remark that “running the discrete-time algorithm (2) with the *best* step-size strategy $\lambda _{t}$ and fixed $\eta$ [...] gives the same (infinite) constant as the corresponding strategy for fixed $\lambda$ and variable $\eta _{t}$” [13, p. 224].

The proof of Theorem 4.2 relies crucially on the following lemma, which provides an explicit estimate for the decay rate of the employed gap functions.

#### Lemma 4.3

If (SMD) is initialized at (0, 0), and Hypotheses (H1)–(H3) hold, then:

16 $$\begin{aligned} g(\bar{X}(t)) \le \frac{\mathcal {D}(h;\mathcal {X})}{\eta (t)S(t)} + \frac{\sigma _{*}^{2}}{2\alpha } \frac{\int _{0}^{t} \lambda ^{2}(s) \eta (s)\,\mathrm{d}s}{S(t)} +\frac{I(t)}{S(t)} \end{aligned}$$

where $$I(t):= \sup _{p\in \mathcal {X}} I_{p}(t)$$ and

17 $$\begin{aligned} I_{p}(t):= \int _{0}^{t} \lambda (s) \langle p - X(s), \sigma (X(s),s) \cdot dW(s)\rangle . \end{aligned}$$

If (MVI) is associated with a convex-concave saddle-point problem as in Example 2.2, we have

$$\begin{aligned} G(\bar{X}^{1}(t),\bar{X}^{2}(t)) \le \frac{\mathcal {D}_{\text{ sp }}}{\eta (t)S(t)} +\frac{\sigma _{*}^{2}}{2\alpha _{\text{ sp }}} \frac{\int _{0}^{t} \lambda ^{2}(s) \eta (s)\,\mathrm{d}s}{S(t)} +\frac{J(t)}{S(t)}, \end{aligned}$$

where we have set $\mathcal {D}_{\text{ sp }}:= \mathcal {D}(h_{1};\mathcal {X}^{1})+\mathcal {D}(h_{2};\mathcal {X}^{2})$, $1/\alpha _{\text{ sp }}:= 1/\alpha _{1} +1/\alpha _{2}$, and $J(t):= \sup _{p^{1}\in \mathcal {X}^{1},p^{2}\in \mathcal {X}^{2}} \{I_{p^{1}}(t) + I_{p^{2}}(t)\}$.

#### Remark 4.1

The initialization assumption in Lemma 4.3 is not crucial: We only make it to simplify the explicit expression (16). If (SMD) is initialized at a different point, the proof of Lemma 4.3 shows that the bound (16) is correct only up to $\mathcal {O}(1/S(t))$. Since all terms in (16) are no faster than $\mathcal {O}(1/S(t))$, initialization plays no role in the proof of Theorem 4.2 below.

#### Proof of Lemma 4.3

Fix some $p\in \mathcal {X}$, and let $H_{p}(t):= \eta (t)^{-1}F(p,\eta (t)Y(t))$, as in the proof of Proposition 3.3. Then, by the weak Itô formula (33) in Sect. 5, we have

18 $$\begin{aligned}&H_{p}(t) \le H_{p}(0) -\int _{0}^{t} \frac{\dot{\eta }(s)}{\eta (s)^{2}} H_{p}(s)\,\mathrm{d}s +\frac{1}{\eta (t)} \int _{0}^{t} \langle X(s)-p,\dot{\eta }(s) Y(s)\rangle \,\mathrm{d}s\nonumber \\&\qquad \qquad \,\,+ \int _{0}^{t} \langle X(s) - p,\,\mathrm{d}Y(s)\rangle + \frac{1}{2\alpha } \int _{0}^{t} \lambda ^{2}(s) \eta (s)||\sigma (X(s),s)||^{2} \,\mathrm{d}s. \end{aligned}$$

To proceed, let

19 $$\begin{aligned} R_{p}(t):= \int _{0}^{t}\lambda (s) \langle v(X(s)),X(s)-p\rangle \,\mathrm{d}s, \end{aligned}$$

so

$$\begin{aligned} \int _{0}^{t} \langle X(s)-p, \,\mathrm{d}Y(s)\rangle&= -\int _{0}^{t} \lambda (s) \langle X(s)-p,v(X(s)) \,\mathrm{d}s + \,\mathrm{d}M(s)\rangle \\&= -R_{p}(t) + I_{p}(t), \end{aligned}$$

with $I_{p}(t)$ given by (17). Then, rearranging and bounding the second term of (18) as in the proof of Proposition 3.3, we obtain

$$\begin{aligned} R_{p}(t)&\le H_{p}(0)-H_{p}(t) + \mathcal {D}(h,\mathcal {X}) \left( \frac{1}{\eta (t)}-\frac{1}{\eta (0)}\right) \\&\quad + I_{p}(t)+ \frac{1}{2\alpha } \int _{0}^{t} \lambda ^{2}(s) \eta (s)||\sigma (X(s),s)||^{2} \,\mathrm{d}s\\&\le H_{p}(0)+ \mathcal {D}(h;\mathcal {X}) \left( \frac{1}{\eta (t)} - \frac{1}{\eta (0)}\right) + I_{p}(t) + \frac{\sigma _{*}^{2}}{2\alpha } \int _{0}^{t} \lambda ^{2}(s) \eta (s) \,\mathrm{d}s. \end{aligned}$$

With (SMD) initialized at $y=0$, Eq. (8) gives $H_{p}(0) \le \mathcal {D}(h;\mathcal {X})/\eta (0)$. Thus, by Jensen’s inequality and the monotonicity of *v*, we get

20 $$\begin{aligned} \langle v(p),\bar{X}(t) - p\rangle&= \frac{1}{S(t)} \int _{0}^{t} \lambda (s) \langle v(p),X(s)-p\rangle \,\mathrm{d}s\nonumber \\&\le \frac{1}{S(t)} \int _{0}^{t} \lambda (s) \langle v(X(s)),X(s)-p\rangle \,\mathrm{d}s= \frac{R_{p}(t)}{S(t)}\nonumber \\&\le \frac{\mathcal {D}(h;\mathcal {X})}{\eta (t)S(t)} + \frac{\sigma _{*}^{2}}{2\alpha } \frac{\int _{0}^{t} \lambda ^{2}(s) \eta (s)\,\mathrm{d}s}{S(t)} +\frac{I_{p}(t)}{S(t)}. \end{aligned}$$

The bound (16) then follows by noting that $g(\bar{X}(t)) = \max _{p\in \mathcal {X}} \langle v(p),\bar{X}(t)-p\rangle$.

Now, assume that (MVI) is associated to a convex-concave saddle-point problem as in Ex. 2.2. As in the proof of Proposition 3.3, we first replicate the analysis above for each component of the problem, and we then sum the two components to get an overall bound for the Nikaido–Isoda gap function *G*. Specifically, applying (20) to (1), we readily get

21 $$\begin{aligned} \int _{0}^{t} \lambda (s) \langle v^{i}(X(s)),X^{i}(s) - p^{i}\rangle \le \frac{\mathcal {D}(h_{i};\mathcal {X}^{i})}{\eta (t) S(t)} + \frac{\sigma _{*}^{2}}{2\alpha ^{i}} \frac{\int _{0}^{t} \lambda ^{2}(s) \eta (s)\,\mathrm{d}s}{S(t)} +\frac{I_{p^{i}}(t)}{S(t)}, \end{aligned}$$

where $i\in \{1,2\}$. Moreover, Jensen’s inequality yields

$$\begin{aligned} U(\bar{X}^{1}(t),p^{2}) - U(p^{1},\bar{X}^{2}(t))&\le \frac{1}{S(t)} \int _{0}^{t} \lambda (s) \left[ U(X^{1}(s),p^{2}) - U(p^{1},X^{2}(s))\right] \,\mathrm{d}s\\&\le \frac{1}{S(t)} \int _{0}^{t} \lambda (s) \langle \nabla _{x^{1}}U(X(s)),X^{1}(s) - p^{1}\rangle \,\mathrm{d}s\\&- \frac{1}{S(t)} \int _{0}^{t} \lambda (s) \langle \nabla _{x^{2}}U(X(s)),X^{2}(s) - p^{2}\rangle \,\mathrm{d}s\\&\le \frac{\mathcal {D}_{\text{ sp }}}{\eta (t)S(t)} +\frac{\sigma _{*}^{2}}{2\alpha _{\text{ sp }}} \frac{\int _{0}^{t} \lambda ^{2}(s) \eta (s)\,\mathrm{d}s}{S(t)} +\frac{I_{p^{1}}(t) + I_{p^{2}}(t)}{S(t)}, \end{aligned}$$

with the last inequality following from (21). Our claim then follows by maximizing over $(p^{1},p^{2})$ and recalling the definition (3) of the Nikaido–Isoda gap function. $\square$

Clearly, the crucial unknown in the bound (16) is the stochastic term *I*(*t*). To obtain convergence of $\bar{X}(t)$ to the solution set of ${{\mathrm{VI}}}(\mathcal {X},v)$, the term *I*(*t*) must grow slower than *S*(*t*). As we show now, this is indeed the case:

#### Proof of Theorem 4.2

By Lemma 4.3 and Remark 4.1, it suffices to show that the term *I*(*t*) grows as $\mathcal {O}(L(t)\sqrt{\log \log L(t)})$ with probability 1. To do so, let $\kappa _{p} := \left[ I_{p}\right]$ denote the quadratic variation of $I_{p}$.^9^ Then, the rules of stochastic calculus yield

$$\begin{aligned} d\kappa _{p}(t)&= dI_{p}(t) \cdot dI_{p}(t)\\&= \lambda ^{2}(t) \sum _{i,j=1}^{n} \sum _{k=1}^{d} (X_{i}(t) - p_{i}) (X_{j}(t) - p_{j}) \sigma _{ik}(X(t),t) \sigma _{jk}(X(t),t) \,\mathrm{d}t\\&\le ||X(t) - p||_{2}^{2} \sigma _{*}^{2}\lambda ^{2}(t)\\&\le {{\mathrm{diam}}}(\mathcal {X})^{2} \sigma _{*}^{2}\lambda ^{2}(t), \end{aligned}$$

where ${{\mathrm{diam}}}(\mathcal {X}):= \max \{||x'-x||_{2}:x,x'\in \mathcal {X}\}$ denotes the Euclidean diameter of $\mathcal {X}$. Hence, for all $t\ge 0$, we get the quadratic variation bound

22 $$\begin{aligned} \kappa _{p}(t) \le {{\mathrm{diam}}}(\mathcal {X})^{2} \sigma _{*}^{2}\int _{0}^{t} \lambda ^{2}(s) \,\mathrm{d}s = \mathcal {O}(L^{2}(t)). \end{aligned}$$

Now, let $\kappa _{p}(\infty ) := \lim _{t\rightarrow \infty } \kappa _{p}(t) \in [0,\infty ]$ and set

$$\begin{aligned} \tau _{p}(s): = {\left\{ \begin{array}{ll} \inf \{t\ge 0:\kappa _{p}(t) > s\}, &{}\quad \text {if } s\le \kappa _{p}(\infty ),\\ \infty , &{}\quad \text {otherwise}. \end{array}\right. } \end{aligned}$$

The process $\tau _{p}(s)$ is finite, nonnegative, nondecreasing and right-continuous on $[0,\kappa _{p}(\infty ))$; moreover, it is easy to check that $\kappa _{p}(\tau _{p}(s)) = s \wedge \kappa _{p}(\infty )$ and $\tau _{p}(\kappa _{p}(t)) = t$ [32, Problem 3.4.5]. Therefore, by the Dambis–Dubins–Schwarz time-change theorem for martingales [32, Theorem. 3.4.6 and Problem. 3.4.7], there exists a standard, one-dimensional Wiener process $(B_{p}(t))_{t\ge 0}$ adapted to a modified filtration $\tilde{\mathcal {F}}_{s} = \mathcal {F}_{\tau _{p}(s)}$ (possibly defined on an extended probability space), and such that $B_{p}(\kappa _{p}(t)) = I_{p}(t)$ for all $t\ge 0$ (except possibly on a ${{\mathrm{\mathbb {P}}}}$-null set). Hence, for all $t>0$, we have

$$\begin{aligned} \frac{I_{p}(t)}{S(t)} = \frac{B_{p}(\kappa _{p}(t))}{S(t)} = \frac{B_{p}(\kappa _{p}(t))}{\sqrt{\kappa _{p}(t) \log \log \kappa _{p}(t)}} \times \frac{\sqrt{\kappa _{p}(t) \log \log \kappa _{p}(t)}}{S(t)}. \end{aligned}$$

By the law of the iterated logarithm [32], the first factor above is bounded almost surely; as for the second, (22) gives $\sqrt{\kappa _{p}(t) \log \log \kappa _{p}(t)} = \mathcal {O}(L(t) \sqrt{\log \log L(t)})$. Thus, combining all of the above, we get

$$\begin{aligned} \frac{I(t)}{S(t)} = \frac{\max _{p\in \mathcal {X}} I_{p}(t)}{S(t)} = \mathcal {O}\left( \frac{L(t) \sqrt{\log \log L(t)}}{S(t)}\right) , \end{aligned}$$

so our claim follows from (16).

To complete our proof, note first that the condition $\lim _{t\rightarrow \infty } \eta (t) S(t) = \infty$ implies that $\lim _{t\rightarrow \infty } S(t) = \infty$ (given that $\eta (t)$ is nonincreasing). Thus, by de l’Hôpital’s rule and the assumption $\lim _{t\rightarrow \infty } \lambda (t) \eta (t) = 0$, we also get $S(t)^{-1} \int _{0}^{t} \lambda ^{2}(s) \eta (s) \,\mathrm{d}s = 0$. Finally, for the last term of (14), consider the following two cases:

1. If $\lim _{t\rightarrow \infty } L(t) < \infty$, we trivially have $\lim _{t\rightarrow \infty } L(t) \sqrt{\log \log L(t)} \big / S(t) = 0$ as well.
2. Otherwise, if $\lim _{t\rightarrow \infty } L(t) = \infty$, de l’Hôpital’s rule readily yields
  $$\begin{aligned} \lim _{t\rightarrow \infty } \frac{L^{2}(t)}{S^{2}(t)} = \lim _{t\rightarrow \infty } \frac{\lambda ^{2}(t)}{2 \lambda (t) S(t)} = \frac{1}{2} \lim _{t\rightarrow \infty } \frac{\lambda (t)}{S(t)} = 0, \end{aligned}$$
  by the boundedness of $\lambda (t)$. Another application of de l’Hôpital’s rule gives
  $$\begin{aligned} \lim _{t\rightarrow \infty } \frac{L^{3}(t)}{S^{2}(t)} = \lim _{t\rightarrow \infty } \frac{(L^{2}(t))^{3/2}}{S^{2}(t)} = \frac{3}{4} \lim _{t\rightarrow \infty } \frac{\lambda ^{2}(t) L(t)}{\lambda (t) S(t)} = \frac{3}{4} \lim _{t\rightarrow \infty } \frac{\lambda (t) L(t)}{S(t)} = 0, \end{aligned}$$
  so
  $$\begin{aligned} \limsup _{t\rightarrow \infty } \frac{L(t)\sqrt{\log \log L(t)}}{S(t)} \le \limsup _{t\rightarrow \infty } \sqrt{\frac{L^{3}(t)}{S^{2}(t)}} = 0. \end{aligned}$$

The above shows that, under the stated assumptions, the RHS of (14) converges to 0 almost surely, implying in turn that $\bar{X}(t)$ converges to the solution set of ${{\mathrm{VI}}}(\mathcal {X},v)$ with probability 1. $\square$

### Large Deviations

In this section, we study the concentration properties of (SMD) in terms of the dual gap function. As in the previous section, we will assume that (SMD) is issued from the initial condition $(s,y)=(0,0)$.

First, recall that for every $p\in \mathcal {X}$ we have the upper bound

$$\begin{aligned} R_{p}(t) \le \frac{\mathcal {D}(h;\mathcal {X})}{\eta (t)} +\frac{\sigma _{*}^{2}}{2\alpha }\int _{0}^{t}\lambda ^{2}(s)\eta (s)\,\mathrm{d}s +I_{p}(t). \end{aligned}$$

with $R_{p}(t)$ and $I_{p}(t)$ defined as in (19) and (17), respectively. Since $I_{p}(t)$ is a continuous martingale starting at 0, we have $\mathbb {E}[I_{p}(t)] = 0$, implying in turn that

$$\begin{aligned} \mathbb {E}[\langle v(p),\bar{X}(t)-p\rangle ] \le \frac{\mathcal {D}(h;\mathcal {X})}{S(t)\eta (t)} + \frac{\sigma _{*}^{2}}{2\alpha S(t)}\int _{0}^{t}\lambda ^{2}(s)\eta (s)\,\mathrm{d}s =\frac{K(t)}{2S(t)}, \end{aligned}$$

where

23 $$\begin{aligned} K(t):= \frac{2\mathcal {D}(h;\mathcal {X})}{\eta (t)}+ \frac{\sigma _{*}^{2}}{\alpha } \int _{0}^{t}\lambda ^{2}(s)\eta (s)\,\mathrm{d}s. \end{aligned}$$

Markov’s inequality therefore implies that

24 $$\begin{aligned} \mathbb {P}\left( \langle v(p),\bar{X}(t)-p\rangle \ge \delta \right) \le \frac{1}{\delta } \frac{K(t)}{2S(t)} \quad \text {for all } \delta >0. \end{aligned}$$

The bound (24) provides a first estimate of the probability of observing a large gap from the solution of (MVI), but because it relies only on Markov’s inequality, it is rather crude. To refine it, we provide below a “large deviations” bound that shows that the ergodic gap process $g(\bar{X}(t))$ is exponentially concentrated around its mean value:

#### Theorem 4.3

Suppose (H1)–(H3) hold, and that (SMD) is started from the initial condition $(s,y)=(0,0)$. Then, for all $\delta >0$ and all $t>0$, we have

25 $$\begin{aligned} \mathbb {P}\left( g(\bar{X}(t))\ge \mathcal {Q}_{0}(t)+\delta \mathcal {Q}_{1}(t)\right) \le \exp (-\delta ^{2}/4), \end{aligned}$$

where

26a $$\begin{aligned} \mathcal {Q}_{0}(t):=\frac{K(t)}{S(t)}, \end{aligned}$$

and

26b $$\begin{aligned} \mathcal {Q}_{1}(t):=\frac{\sqrt{\kappa }\sigma _{*}{{\mathrm{diam}}}(\mathcal {X})L(t)}{S(t)}, \end{aligned}$$

with $\kappa >0$ a positive constant depending only on $\mathcal {X}$ and $||\cdot ||$.

The concentration bound (25) can also be formulated as follows:

#### Corollary 4.2

With notation and assumptions as in Theorem 4.3, we have

$$\begin{aligned} g(\bar{X}(t))&\le \mathcal {Q}_{0}(t) + 2 \mathcal {Q}_{1}(t) \sqrt{\log (1/\delta )}\\&= \mathcal {O}\left( \frac{1}{\eta (t) S(t)}\right) + \mathcal {O}\left( \frac{\int _{0}^{t} \lambda ^{2}(s) \eta (s)\,\mathrm{d}s}{S(t)}\right) + \mathcal {O}\left( \frac{L(t) \sqrt{\log (1/\delta )}}{S(t)}\right) , \end{aligned}$$

with probability at least $1-\delta$. In particular, if (SMD) is run with parameters $\lambda (t) = (1+t)^{-a}$ and $\eta (t) = (1+t)^{-b}$ for some $a,b\in [0,1]$, we have

$$\begin{aligned} g(\bar{X}(t)) = \mathcal {O}\left( t^{a+b-1}\right) + \mathcal {O}\left( t^{-a-b}\right) + \mathcal {O}\left( t^{-1/2}\right) , \end{aligned}$$

with arbitrarily high probability.

To prove Theorem 4.3, define first the auxiliary processes

$$\begin{aligned} Z(t):= \int _{0}^{t}\lambda (s)\sigma (X(s),s)\,\mathrm{d}W(s) \quad \text {and} \quad P(t):= Q(\eta (t)Z(t)). \end{aligned}$$

We then have:

#### Lemma 4.4

For all $p\in \mathcal {X}$ we have

27 $$\begin{aligned} \int _{0}^{t} \lambda (s)\langle p-P(s),\sigma (X(s),s)\,\mathrm{d}W(s)\rangle \le \frac{\mathcal {D}(h;\mathcal {X})}{\eta (t)} + \frac{\sigma _{*}^{2}}{2\alpha }\int _{0}^{t}\lambda ^{2}(s)\eta (s)\,\mathrm{d}s. \end{aligned}$$

#### Proof

The proof follows the same lines as Lemma 4.3. Specifically, given a reference point $p\in \mathcal {X}$, define the process $\tilde{H}_{p}(t):= \frac{1}{\eta (t)} F(p,\eta (t)Z(t))$. Then, by the weak Itô formula (33) in Sect. 5, we have

$$\begin{aligned} \tilde{H}_{p}(t)&\le \tilde{H}_{p}(0)\\&\quad -\int _{0}^{t} \frac{\dot{\eta }(s)}{\eta (s)^{2}}\tilde{H}_{p}(s)\,\mathrm{d}s+\frac{1}{\eta (t)} \int _{0}^{t} \langle \xi (s)-p,\dot{\eta }(s)Z(s)\rangle \,\mathrm{d}s\\&\quad + \int _{0}^{t} \langle P(s) - p,\,\mathrm{d}Z(s)\rangle + \frac{1}{2\alpha } \int _{0}^{t} \lambda ^{2}(s) \eta (s)||\sigma (X(s),s)||^{2} \,\mathrm{d}s\\&\le -\int _{0}^{t}\frac{\dot{\eta }(s)}{\eta (s)}[h(p)-h(P(s))]\,\mathrm{d}s\\&\quad + \int _{0}^{t}\lambda (s)\langle P(s)-p,\sigma (X(s),s)\,\mathrm{d}W(s)\rangle + \frac{\sigma _{*}^{2}}{2\alpha } \int _{0}^{t} \lambda ^{2}(s)\eta (s) \,\mathrm{d}s. \end{aligned}$$

We thus get

$$\begin{aligned} \int _{0}^{t}\lambda (s)\langle p-P(s),\sigma (X(s),s)\,\mathrm{d}W(s)\rangle&\le \tilde{H}_{p}(0) + \mathcal {D}(h;\mathcal {X}) \, \left( \frac{1}{\eta (t)}-\frac{1}{\eta (0)}\right) \\&\quad + \int _{0}^{t}\frac{\lambda ^{2}(s)\eta (s)}{2\alpha }\sigma _{*}^{2}\,\mathrm{d}s\\&\le \frac{\mathcal {D}(h;\mathcal {X})}{\eta (t)} + \int _{0}^{t}\frac{\lambda ^{2}(s)\eta (s)}{2\alpha }\sigma _{*}^{2}\,\mathrm{d}s, \end{aligned}$$

as claimed. $$\square $$

We are now ready to establish our large deviations principle for (SMD):

#### Proof of Theorem 4.3

For $p\in \mathcal {X}$ and $t>0$ fixed, we have

$$\begin{aligned} R_{p}(t)&\le \frac{\mathcal {D}(h;\mathcal {X})}{\eta (t)} + \frac{\sigma _{*}^{2}}{2\alpha }\int _{0}^{t}\lambda ^{2}(s)\eta (s)\,\mathrm{d}s + \int _{0}^{t}\lambda (s)\langle p-X(s),\sigma (X(s),s)\,\mathrm{d}W(s)\rangle \\&= \frac{\mathcal {D}(h;\mathcal {X})}{\eta (t)} + \frac{\sigma _{*}^{2}}{2\alpha }\int _{0}^{t}\lambda ^{2}(s)\eta (s)\,\mathrm{d}s + \int _{0}^{t}\lambda (s)\langle p-P(s),\sigma (X(s),s)\,\mathrm{d}W(s)\rangle \\&\quad + \int _{0}^{t}\lambda (s)\langle P(s)-X(s),\sigma (X(s),s)\,\mathrm{d}W(s)\rangle \\&\le \frac{2\mathcal {D}(h;\mathcal {X})}{\eta (t)} + \frac{\sigma _{*}^{2}}{\alpha }\int _{0}^{t}\lambda ^{2}(s)\eta (s)\,\mathrm{d}s + \int _{0}^{t}\langle P(s)-X(s),\sigma (X(s),s)\,\mathrm{d}W(s)\rangle , \end{aligned}$$

where we used (27) to obtain the last inequality. To proceed, let

$$\begin{aligned} \varDelta (t):=\int _{0}^{t} \lambda (s)\langle P(s)-X(s),\sigma (X(s),s) \,\mathrm{d}W(s)\rangle . \end{aligned}$$

The process $\varDelta (t)$ is a continuous martingale starting at 0 which is almost surely bounded in $L^{2}$, thus providing an upper bound for $R_{p}(t)$ which is independent of the reference point $p\in \mathcal {X}$. Indeed, recalling the definition (23) of *K*(*t*), we see that

$$\begin{aligned} R_{p}(t)\le K(t)+\varDelta (t), \end{aligned}$$

so

$$\begin{aligned} g(\bar{X}(t)) \le \frac{K(t) +\varDelta (t)}{S(t)} \quad \text {for all } t>0. \end{aligned}$$

In turn, this implies that for all $\varepsilon ,t>0$,

$$\begin{aligned} \mathbb {P}\left( g(\bar{X}(t))\ge \varepsilon \right) \le \mathbb {P}\left( \varDelta (t)\ge \varepsilon S(t)-K(t)\right) \quad \text {for all } \varepsilon ,t>0. \end{aligned}$$

To prove the theorem, we are left to bound the right-hand side of the above expression. To that end, letting $\rho (t):= [\varDelta (t),\varDelta (t)]$ denote the quadratic variation of $\varDelta (t)$, the Cauchy–Schwarz inequality readily gives

$$\begin{aligned} \mathbb {E}[\exp (\theta \varDelta (t))]&= \mathbb {E}[\exp (\theta \varDelta (t)-b\rho (t))\exp (b\rho (t))]\\&\le \sqrt{\mathbb {E}[\exp (2\theta \varDelta (t)-2b\rho (t))]} \sqrt{\mathbb {E}[\exp (2b\rho (t))]}. \end{aligned}$$

Setting $b=\theta ^{2}$, the expressions inside the first expected value is just the stochastic exponential of the process $2\theta \varDelta (t)$. Moreover, a straightforward calculation shows that

$$\begin{aligned} \rho (t)&= \int _{0}^{t} \lambda ^{2}(s) ||\sigma (X(s),s) \cdot (P(s)-X(s))||_{2}^{2} \,\mathrm{d}s\\&\le \kappa \int _{0}^{t}\lambda ^{2}(s)||\sigma (X(s),s)||^{2}||P(s)-X(s)||^{2}\,\mathrm{d}s\\&\le \kappa \sigma _{*}^{2}{{\mathrm{diam}}}(\mathcal {X})^{2}L^{2}(t), \end{aligned}$$

where $\kappa >0$ is a universal constant accounting for the equivalence of the Euclidean norm $||\cdot ||_{2}$ and the primal norm $||\cdot ||$ on $\mathcal {X}$. The above implies that $\rho (t)$ is bounded over every compact interval, showing that Novikov’s condition is satisfied (see, e.g., [32]). We conclude that the process $\exp (2\theta \varDelta (t)-2\theta ^{2}\rho (t))$ is a true martingale with expected value 1. Hence, letting $\varphi (t):=\kappa \sigma _{*}^{2}{{\mathrm{diam}}}(\mathcal {X})^{2}L^{2}(t)$, we get

28 $$\begin{aligned} \mathbb {E}[\exp (\theta \varDelta (t))] \le \sqrt{\mathbb {E}[\exp (2\theta ^{2}\rho (t)]} \le \exp (\theta ^{2}\varphi (t)). \end{aligned}$$

Combining all of the above, we see that for all $a>0$

$$\begin{aligned} \begin{array}{lll} \mathbb {P}\left( \varDelta (t)\ge a\right) &{}= \mathbb {P}\left( \exp (\theta \varDelta (t))\ge \exp (\theta a)\right) &{}\\ &{}\le \exp (-a\theta ) \mathbb {E}[\exp (\theta \varDelta (t))]&{}\quad {\mathrm{(Markov}}~{\mathrm{inequality)}}\\ &{}=\exp (-a\theta + \theta ^{2} \varphi (t)) \quad \text {for all } a>0,&{}\\ \end{array} \end{aligned}$$

with the last line following from (28). Minimizing the above with respect to $\theta$ then gives

$$\begin{aligned} \mathbb {P}\left( \varDelta (t)\ge a\right) \le \exp \left( -\frac{a^{2}}{4\varphi (t)}\right) . \end{aligned}$$

Hence, by unrolling Eqs. (26a) and (26b), we finally obtain the bound

$$\begin{aligned} \mathbb {P}\left( g(\bar{X}(t))\ge \mathcal {Q}_{0}(t)+\delta \mathcal {Q}_{1}(t)\right)&\le \mathbb {P}\left( \varDelta (t)\ge \mathcal {Q}_{0}(t)S(t) + \delta \mathcal {Q}_{1}(t)S(t)-K(t)\right) \\&\le \mathbb {P}\left( \varDelta (t)\ge \delta \sqrt{\varphi (t)}\right) \\&\le \exp (-\delta ^{2}/4), \end{aligned}$$

for all $\delta >0$, as claimed. $\square$

## Conclusions

This paper examined a continuous-time dynamical system for solving monotone variational inequality problems with random inputs. The key element of our analysis is the identification of a energy-type function, which allows us to prove ergodic convergence of generated trajectories in the deterministic as well as in the stochastic case. Future research should extend the present work in the following dimensions. First, it is not clear yet how the continuous-time method will help us in the derivation of a consistent numerical scheme. A naive Euler discretization might potentially lead to a loss in speed of convergence (see [2]). Second, it is of great interest to relax the monotonicity assumption we made on the involved operator. We are currently investigating these extensions. Third, it is of interest to consider different noise models as well. In particular, it would be interesting to know how the results derived in this paper change when the stochastic perturbation comes from a jump Markov process, or more generally, a Lévy process. This extension would likely need new techniques, and we regard this as an important contribution for future work.

## Appendix A: Results from Convex Analysis

In this appendix, we collect some simple facts on the analysis of convex differentiable functions with Lipschitz continuous gradients. Denote by $\mathbf C ^{1,1}_{L}(\mathbb {R}^n)$ the totality of such functions, with *L* being the Lipschitz constant of the gradient mapping $\nabla \psi$:

$$\begin{aligned} ||\psi (y+\delta )-\psi (y)||_{*}\le L||\delta ||_{*}\qquad \forall y,\delta \in \mathbb {R}^n. \end{aligned}$$

### Proposition A.1

Let $\psi \in \mathbf C ^{1,1}_{L}(\mathbb {R}^n)$ be convex. Then, $\psi$ is almost everywhere twice differentiable with Hessian $\nabla ^{2}\psi$ and

$$\begin{aligned} 0\le \nabla ^{2}\psi (y)\le L{{\mathrm{Id}}}.\quad {\mathsf {Leb}}-\text {a.e.}. \end{aligned}$$

### Proof

For every $\psi \in \mathbf C ^{1,1}_{L}(\mathbb {R}^n)$, the well-known descent lemma ([33], Theorem 2.1.5) implies that

29 $$\begin{aligned} \psi (y+\delta )\le \psi (y)+\langle \nabla \psi (y),\delta \rangle +\frac{L}{2}||\delta ||_{*}^{2} \qquad \forall y,\delta \in \mathbb {R}^n. \end{aligned}$$

By Alexandrov’s theorem (see, e.g., [34], Lemma 6.6), it follows that $\psi$ is ${\mathsf {Leb}}$-almost everywhere twice differentiable. Hence, there exists a measurable set $\varLambda$ such that ${\mathsf {Leb}}(\varLambda )=0$, and for all $\bar{y}\in \mathbb {R}^n\setminus \varLambda$ there exists $(p,P)\in \mathbb {R}^n\times \mathbb {R}^{n\times n}_{sym}$ such that

30 $$\begin{aligned} \psi (\bar{y}+y)=\psi (\bar{y})+\langle p,y\rangle +\frac{1}{2}\langle Py,y\rangle +\theta (\bar{y},y), \end{aligned}$$

where $\lim _{||y||_{*}\rightarrow 0}\frac{\theta (\bar{y},y)}{||y||^{2}_{*}}=0$. We have $p=\nabla \psi (\bar{y})$ and identify *P* with the a.e. defined Hessian $\nabla ^{2}\psi (\bar{y})$. On the other hand, convexity implies

31 $$\begin{aligned} \psi (\bar{y}+y)\ge \psi (\bar{y})+\langle \nabla \psi (\bar{y}),y\rangle \qquad \forall \bar{y},y\in \mathbb {R}^n. \end{aligned}$$

Choosing $y=t e$, where $e\in \mathbb {R}^n$ is an arbitrary $||\cdot ||_{*}$-unit vector and $t>0$, it follows

$$\begin{aligned} -\frac{1}{t^{2}}\theta (\bar{y},te)&{\mathop {\le }\limits ^{\mathrm{(31)}}}\frac{1}{t^{2}}[\psi (\bar{y}+te)-\psi (\bar{y}) -\langle \nabla \psi (\bar{y}),te\rangle ]-\frac{1}{t^{2}}\theta (\bar{y},te)\\&{\mathop {=}\limits ^{\mathrm{(30)}}}\frac{1}{2}\langle \nabla ^{2}\psi (\bar{y})e,e\rangle \\&{\mathop {\le }\limits ^{\mathrm{(29)}}}\frac{L}{2}-\frac{1}{t^{2}}\theta (\bar{y},te). \end{aligned}$$

Letting $t\rightarrow 0^{+}$ we get

32 $$\begin{aligned} 0\le \frac{1}{2}\langle \nabla ^{2}\psi (\bar{y})e,e\rangle \le \frac{L}{2}\qquad \forall \bar{y}\in \mathbb {R}^n\setminus \varLambda , \end{aligned}$$

which implies $$\nabla ^{2}\psi (\bar{y})\le L{{\mathrm{Id}}}$$. $$\square $$

## Appendix B: Results from Stochastic Analysis

The following result is the generalized Itô formula, used in the main text.

### Proposition B.1

Let *Y* be an Itô process in $\mathbb {R}^n$ of the form

$$\begin{aligned} Y_{t}=Y_{0}+\int _{0}^{t}F_{s}\,\mathrm{d}s+\int _{0}^{t}G_{s}\,\mathrm{d}W(s). \end{aligned}$$

Let $\psi \in \mathbf C ^{1,1}_{L}(\mathbb {R}^n)$ be convex. Then, for all $t\ge 0$, we have

$$\begin{aligned} \psi (Y_{t})\le \psi (Y_{0})+\int _{0}^{t}\langle \nabla \psi (Y_{s}),\,\mathrm{d}Y_{s}\rangle +\frac{L}{2}\int _{0}^{t}||G_{s}||^{2}\,\mathrm{d}s \end{aligned}$$

### Proof

Since $\psi \in \mathbf C ^{1,1}_{L}(\mathbb {R}^n)$ is convex, Proposition A.1 shows that $\psi$ is almost everywhere twice differentiable with Hessian $\nabla ^{2}\psi$. Furthermore, this Hessian matrix satisfies $0\le \nabla ^{2}\psi (y)\le L{{\mathrm{Id}}}$, for all $y\in \mathbb {R}^n$ outside a set of Lebesgue measure 0.

Introduce the mollifier

$$\begin{aligned} \rho (u):=\left\{ \begin{array}{lr} c\exp \left( \frac{-1}{1-||u||_{*}^{2}}\right) , &{} \text { if }||u||_{*}<1,\\ 0, &{} \text {if }||u||_{*}\ge 1. \end{array}\right. \end{aligned}$$

Choose the constant $c>0$ so that $\int _{\mathbb {R}^n}\rho (u)\,\mathrm{d}u=1$. For every $\varepsilon >0$ define

$$\begin{aligned} \rho _{\varepsilon }(u)&:=\varepsilon ^{-n}\rho (u/\varepsilon ),\\ \psi _{\varepsilon }(y)&:=\psi \circledast \rho _{\varepsilon }(y):=\int _{\mathbb {R}^n}\psi (y-u)\rho _{\varepsilon }(u)\,\mathrm{d}u. \end{aligned}$$

Then, $\psi _{\varepsilon }\in \mathbf C ^{\infty }(\mathbb {R}^n)$ and the standard form of Itô’s formula gives us

$$\begin{aligned} \psi _{\varepsilon }(Y_{t})&=\psi _{\varepsilon }(Y_{s})+\int _{s}^{t}\langle \nabla \psi _{\varepsilon }(Y_{r}),\,\mathrm{d}Y_{r}\rangle +\frac{1}{2}\int _{s}^{t}{{\mathrm{tr}}}\left[ \nabla ^{2}\psi _{\varepsilon } (Y_{r})G_{r}G_{r}^{\top }\right] \,\mathrm{d}r\\&=\psi _{\varepsilon }(Y_{s})+\int _{s}^{t}\left\langle \int _{\mathbb {R}^n} \nabla \psi (z)\rho _{\varepsilon }(Y_{r}-z)\,\mathrm{d}z,\,\mathrm{d}Y_{r}\right\rangle \\&\quad +\frac{1}{2}\int _{s}^{t}\int _{\mathbb {R}^n}{{\mathrm{tr}}}\left[ \nabla ^{2}\psi (z)G_{r}G_{r}^{\top }\right] \rho _{\varepsilon }(Y_{r}-z)\,\mathrm{d}r\,\mathrm{d}z. \end{aligned}$$

Since ${{\mathrm{tr}}}(\nabla ^{2}\psi (z)G_{r}G_{r}^{\top })\le L||G_{r}||^{2}$, we get

$$\begin{aligned} \psi _{\varepsilon }(Y_{t})&\le \psi _{\varepsilon }(Y_{s})+\int _{s}^{t} \left\langle \int _{\mathbb {R}^n}\nabla \psi (z)\rho _{\varepsilon }(Y_{r}-z)\,\mathrm{d}z,\,\mathrm{d}Y_{r}\right\rangle +\frac{L}{2}\int _{s}^{t}||G_{r}||^{2}\,\mathrm{d}r. \end{aligned}$$

Letting $\varepsilon \downarrow 0$, using the uniform convergence of the involved data, proves the result. $\square$

Applying this result to the dual process of (SMD) and using (32), gives for $F_{s}=A(s,Y(s,y))$ and $G_{s}=B(s,Y(s,y))$, the following version of the generalized Itô rule:

33 $$\begin{aligned} \psi (Y_{t})\le \psi (Y_{0})+\int _{0}^{t}\langle \nabla \psi (Y_{s}),\,\mathrm{d}Y_{s}\rangle \,\mathrm{d}s+\frac{1}{2\alpha }\int _{0}^{t}||B(s,Y(s,y))||^{2}\,\mathrm{d}s \end{aligned}$$

## Footnotes

1 Equivalence is understood in the sense that $x_{*}\in \mathcal {X}$ solves (MVI), if and only if it solves (SVI). See Proposition 3.1 in [7], and Proposition 3 in [8], which treats also extensions to Vector Variational Inequalities.

2 In the deterministic case, similar results are to be contrasted with the use of Gauss–Seidel methods for solving Variational and (generalized) Nash Equilibrium problems; for a survey, see [17, 18].

3 Interestingly, the corresponding rate for the deterministic (noise-free) dynamics is $\mathcal {O}(1/t)$, indicating a substantial drop from the deterministic to the stochastic regime. This drop is consistent with the black-box convergence rate of Mirror Descent in (stochastic) VIs [13] and is due to the second-order Itô correction that appears in the stochastic case.

4 The usual initialization is $y_{0}=0$, $x_{0} = Q(0) = {\mathop {{{\mathrm{argmin}}}}\limits _{x\in \mathcal {X}}} h(x)$, but other initializations are possible.

5 The name “dual averaging” alludes to the choice $\lambda _{t} = 1$, $\eta _{t} = 1/t$: under this choice of parameters, $x_{t}$ is a mirror projection of the “dual average” $y_{t} = t^{-1} \sum _{s=0}^{t-1} v(x_{s})$.

6 We tacitly assume here that the filtration $(\mathcal {F}_{t})_{t\ge 0}$ satisfies the usual conditions of right continuity and completeness, and carries a standard *d*-dimensional Wiener process $(W(t))_{t\ge 0}$.

7 In fact, even faster convergence can be guaranteed if (MD) is run with *increasing* $\lambda (t)$. In that case however, well posedness is not immediately guaranteed, so we do not consider increasing $\lambda$ here.

8 More precisely, we write $f(x) = \tilde{\mathcal {O}}(g(x))$ when $f(x) = \mathcal {O}(g(x) \log ^{k}g(x))$ for some $k>0$.

9 Recall here that the quadratic variation of a stochastic process *M*(*t*) is the continuous increasing and progressively measurable process, defined as $\left[ M(t)\right] = \lim _{|\varPi |\rightarrow 0} \sum _{1\le j \le k} (M(t_{j}) - M(t_{j-1}))^{2}$, where the limit is taken over all partitions $\varPi = \{t_{0} = 0< t_{1}< \cdots < t_{k} = t\}$ of [0, *t*] with mesh $|\varPi | \equiv \max _{j} |t_{j} - t_{j-1}| \rightarrow 0$ [32].
